# Do major health shocks affect the interconnectedness of E-commerce and electronic payment markets? a regional analysis

**DOI:** 10.1007/s10660-022-09669-y

**Published:** 2023-01-13

**Authors:** Samet Gunay, Catherine Prentice, Mohamed Sraieb

**Affiliations:** 1 Finance Department, American University of the Middle East, Kuwait City, Kuwait; 2 Faculty of Business, Education, Law and Arts, University of Southern Queensland, Springfield Central, Australia; 3 Griffith University, Brisbane, Australia; 4 Staffordshire Business School, Staffordshire University, Stoke-on-Trent, UK

**Keywords:** The COVID19 pandemic, E-commerce index, Electronic payment index

## Abstract

In view of the recent pandemic and its associated impact, this study examines the relationship between e-commerce and mobile/electronic payment markets by utilizing two indices as proxies of these market developments. The study employed DCC-GARCH modeling, Hacker–Hatemi bootstrap causality test, Diebold–Yilmaz volatility spillover analysis and a volatility modeling incorporating COVID19 related death statistics of three regions: America, Europe and Asia. The results show that while the two markets display very high time-varying correlations across years, a significant causal relationship is only found during the pandemic. Causality runs from the mobile/electronic payment index to the e-commerce index. Volatility spillover analysis further supports this finding. Interestingly, the mobile/electronic payment index tends to become a net volatility transmitter in the pandemic period. When we incorporate regional COVID19 statistics on cases and deaths in the volatility modeling of the e-commerce index, we find that only COVID19 deaths in Europe have a significant effect on e-commerce returns. This result may be rationalized by the relative tightness of the e-commerce market in Europe compared to America and Asia. Likewise, demographic characteristics might be another potential driver for our findings.

## Introduction

The effects of major health shocks on economic and financial relationships have increasingly attracted the attention of policymakers, researchers and the public since the outbreak in late 2019. The external shock induced by the lethal virus are found to change the intensity, shape and direction of connections between markets. This health crisis has generated a stream of research focused on evaluating the effects on consumers, firms and markets behavior as well as on the connections between markets. In particular, e-commerce and mobile/electronic payment have risen rapidly during this pandemic. In view of this phenomenon, this paper empirically investigates the extent to which a major health shock such as the COVID19 can shape the interconnection between e-commerce and mobile/electronic payment markets.

E-commerce has progressively become a major trend for business exchanges. The recorded sales (retail) were approximately 4.9 trillion U.S.$ globally in 2021, progressed by 16.8% compared to the figure of 2020 and are expected to increase to 5,545 trillion U.S.$ in 2022 [56]. E-commerce has a substantial impact on the economy, growth, the nature of goods being traded and costs and profits, as well as the payment tools being used. E-commerce is a driving force for economic growth. By its very nature, barriers to entry are lower than in the traditional commerce channels. This opens the door for firms of all sizes to compete and enlarge their market shares. The profit appeal generates incentives for entrepreneurs and encourages businesses to enhance digitalization further, which in turn, drives growth and innovation and attracts more consumers to promote e-commerce.

The evolution of e-commerce over time has induced significant changes in consumer behavior. Among the most salient features of these changes, there is a clear tendency to digitalization and the use of online purchases along with electronic payments. This pattern seems to be uniform across most countries and regions, but disparities in strength and intensity are reported. These changes have been magnified by the health shock occurring with the COVID19 pandemic. The preventive measures such as social distancing measures, the curfews and lockdowns undertaken facilitated a transition of consumer behavior towards technology-based purchase experiences and pushed a large increase in online purchases. The guiding line is that technology and national regulations on the one side (macro factors) and agents' risk aversion (micro factors) have eased this transition worldwide. Nevertheless, the progress in the spread of e-commerce was uneven among countries. Those that were initially lagging behind progressed the most. Expectedly, this pattern was associated with a surge in digital payment instruments, including online and contactless means. The rate of progress in the use of digital payments was unprecedented across all times [3].

Consistent with the foregoing discussion, this paper investigates the changes driven by a major health shock on e-commerce and mobile/electronic payments. Understanding the patterns and interactions between these markets has important implications for e-commerce researchers and the relevant practitioners. Using COVID19 as a natural experiment, our findings suggest that the health shock propelled e-commerce and mobile/electronic payments on a higher path. This effect seems to be driven by changes in consumer behavior, and particularly their risk aversion and uncertainties characterizing some phases of the pandemic. Interestingly, we find evidence that the interaction between e-commerce and mobile/electronic payment persisted even after the pandemic surge. However, time and space disparities characterize these changes. On the one hand, only death, not cases, in Europe and other regions seems significantly affected the global relationship between mobile/electronic payment and e-commerce markets. Potential explanations of these results are likely to use different dimensions including initial economic conditions in a country (revenues distribution, poverty, unemployment and size of the informal sectors along with the existence of social safety nets). The initial conditions would affect consumers' revenue and, therefore their online purchase behavior in terms of value and frequency. Habit, confidence, expectation about the future, as well as attitude toward uncertainty and risk are also important drivers. Demographic conditions have also a role to play in the explanation. Standard demand theory stipulates that the composition of the population in terms of size, gender and age distribution directly affect consumer behaviors and the nature of goods being purchased. During a major external shock, the list of factors affecting consumers' behavior should include the nature of government responses to the surge of the shock. So, governments reacted promptly by imposing anti-shock measures. Others leaned to pressure and adopted a ''wait and see'' attitude. Among those that reacted swiftly, some have imposed very strict constraining measures. While others imposed more flexible measures. Government policies also differed in speed and spread of countermeasures such as vaccine campaigns. All of these factors combine and interact to give a rationale for the patterns observed.

The following section discusses the relevant literature and presents the research questions, followed by outlining the methodology. The data analysis and results are presented. The discussion and the implications of the research findings conclude this study.

## Literature review

As reported by Gunay et al. [25] in a sectorial analysis of Australia, when facing a major external shock (war, wildfires, earthquakes, floods, pandemics etc.), consumers' behavior tends to change particularly in the short run. Researchers (e.g., [19, 33, 41, 45, 48]) examine the changes in consumer behavior during economic crises, and indicate that business cycle downturns during financial crises push toward a consumption behavior as a result of economic, environmental and social responsibility concerns. The guiding line for major shocks, in terms of consumer responses, is that recession drives up uncertainty of agents about income and employment which exacerbates their risk-aversion and ultimately result in consumption smoothing behavior [35]. A tendency for simplicity intensifies during stress periods in opposition to the overwhelming multiplicity of choices, along with a tendency to reinforce consumer switching behavior facilitated by improved information technology and high internet penetration [19, 38].

Consumer switching behavior also features the effects of the COVID19 pandemic on consumers. Nevertheless, the COVID19 pandemic distinguishes itself from other crises due to its persistence and its occurrence at a time characterized by high technological advances. The preventive measures undertaken internationally led to a drastic increase in online purchases and facilitated a transition of consumer behavior towards technology-based purchase experiences [7]. These results are resonated with Jamunadevi’s et al. [29] findings that the pandemic of COVID19 induced a significant change of consumer purchase behavior. Valaskova et al. [54] investigate the changes in purchasing patterns following the COVID19 pandemic in Slovakia, using a Pearson's chi-square test and simple and multivariate correspondence analysis to identify the impact of the new purchasing driven by the pandemic. The authors find that consumers' age, income level, and job sectors determine the effects of the pandemic on purchasing behavior. Jo et al. [31] reported the similar findings, by investigating the changes in consumption patterns by industry type and activities related to travel, education and luxury goods.

In terms of purchasing methods, the incentives to mitigate the risk of infection lifted online purchases. This pattern was consolidated by the government restrictions and social distancing requirements [16, 39]. The drivers of this increasing importance of online purchasing practices have been examined by Gu et al. [22]. The paper uses correlation analysis to investigate the drivers of consumers' online purchases in ten countries. The findings suggest that an increased experience and the awareness and readiness in decision making tend to be the important determinant of online purchase. Alfonso et al. [3] use official national statistics on online retail sales as well as private sector market survey sources (Statista, and surveys from IBM, GlobalWebIndex, Contentsquare, etc.) for a large set of countries. The main findings confirm the claims that (i) e-commerce expanded worldwide during the pandemic, (ii) This expansion was not homogenous across sectors and across stages of the pandemic, and (iii) e-commerce expansion was uneven across countries. Those countries initially lagging in terms of e-commerce progressed the most. A common conjecture in the literature is that this pattern emphasizing the progress in electronic payment, which is reinforced by widespread technological advances, seems to be permanent [20, 40, 47, 50].

This surge in e-commerce following the COVID19 pandemic is supported by a change in the perception of the relative costs and benefits of payment methods. E-payments have reduced health risks compared to cash and the increased efforts toward a more user-friendly experience when using E-payments reinforced their attractiveness. Thus, consumers' preferences for cash decreased in favor of E-payment methods [4, 32]. The evolution of this tendency seems to be supported by technological progress. In general, [20] find that the COVID19 pandemic has affected the public's willingness to accept 5G base stations. The pandemic can be seen as a contextual factor that shaped people's acceptance of this technology by affecting the balance between their perception of the usefulness and ease of use of that technology on the one hand and people's perceived risk associated with the 5G base stations on the other hand. It could be inferred that a higher acceptance of the 5G technology would facilitate further development of e-commerce and electronic payments. Despite abundant literature on consumer payment behavior [5, 27, 55, 57], the effects of disruptive events have received very limited attention in the relevant literature. Nevertheless, a few researchers [10, 13, 22, 36] indicate that consumers shifted away from cash for their purchases although consumers increased their cash holding as a store of value. Additionally, Jílková and Králová [30] examine the drivers of the change in the purchase behavior of consumers during the COVID19 pandemic. They find that shifts to digital spending are uneven across sectors. However, purchasing frequency is uniformly higher during the pandemic. Kawasaki et al. [34] focus on consumers’ intentions and investigate the factors that lead to the changes in these intentions toward using e-commerce during and after the COVID19 pandemic in Japan. In the same vein, Campisi et al. [8] conducted a statistical assessment of the effect of COVID19 on e-commerce in some European countries. Alcedo et al. [2] extend the analysis to 47 economies and 26 industries and describe stylized facts for the effects of e-commerce on consumer spending using data on credit card transactions during the COVID19 pandemic.

Drawing on their studies, our paper aims to fill the gap by investigating (i) the relationship between the pandemic and adoption of e-commerce; (ii) the relationship between the pandemic and adoption of mobile/electronic payments; and (iii) the relationship between e-commerce and mobile/electronic payment markets during the pandemic.

## Methodology

### Theoretical framework

This study employed two fundamental models: GARCH and VAR methodology. As Engle [18] advocated, dynamic conditional correlations analysis constructed on the GARCH model allows to examine time-varying correlations. Therefore, we use GARCH modeling to investigate the time-varying relationships of our markets. One of the major advantages of GARCH is that it would enable us to observe regional consumer reactions on the mean and variance of the equations of the model. Prior to the GARCH model, we test the causal relationship between these two markets through the methodology of Hacker and Hatemi-J [26] to determine the direction of interactions between the mobile/electronic payment and e-commerce companies. To measure this interaction, we use the Solactive e-Commerce Index (Solec) as a proxy for e-commerce. Mobile/electronic payments are represented by the Solactive Electronic Payment Index (Solepmt). Both indexes are obtained online from Solactive- a German Index Engineering company. More precisely, Solec index tracks changes in the share price of companies operating in the e-commerce market. According to Solactive company, this market includes “firms that operate E-commerce platforms, provide E-commerce software, analytics or services, and/or primarily sell goods and services online and generate the majority of their overall revenue from online retail”. Similarly, Solepmt index traces price changes in shares of “firms that generate the majority of their revenues in the area of mobile payments and electronic payment processing”. Example of such companies include Alibaba, PayPal, Square, etc. [49].

The model is built on the vector autoregression methodology and does not necessitate the determination of the order of the integration and test of cointegration properties of the series. Our results also build on a volatility spillover analysis. More precisely, we use the Diebold–Yilmaz volatility spillover analysis, which also employs vector autoregression methodology, as a complementary and confirmation step to the findings based on the former causality tests. Finally, we proceed with the GARCH analysis to test the impact on the mobile/electronic payments market of our independent variables and the regional COVID19 cases and deaths statistics. All these independent variables are tested for their impacts on the mean and variance equations of the GARCH model. A detailed description of the econometric models used is presented below.

### GARCH and DCC-GARCH

The GARCH model introduced by Engle [18] allows the examination of time-varying correlations and produces sensible empirical outcomes. The method requires two models' estimation: a series of univariate GARCH models and the dynamic conditional correlations. As demonstrated by Engle [17], let ${\mathrm{y}}_{\mathrm{t}}$ denotes a stochastic process for the information set $\Psi$, a univariate GARCH model^1^ can be written as in Eq. 2.

1 $${\mathrm{y}}_{\mathrm{t}}=\upmu +{\upepsilon }_{\mathrm{t}} {\upepsilon }_{\mathrm{t}} |{\Psi }_{\mathrm{t}-1}\sim \mathcal{N}\left(0 ,{\mathrm{h}}_{\mathrm{t}}\right)$$

2 $${\mathrm{h}}_{\mathrm{t}}=\upnu + \sum_{\mathrm{i}=1}^{\mathrm{q}}{\mathrm{\alpha }}_{\mathrm{i}}{\upepsilon }_{\mathrm{t}-\mathrm{i}}^{2}+\sum_{\mathrm{i}=1}^{\mathrm{p}}{\upbeta }_{\mathrm{i}}{\mathrm{h}}_{\mathrm{t}-\mathrm{i}}$$

where $\mathrm{p}\ge 0,\mathrm{ q}>0,\upnu >0,{\mathrm{\alpha }}_{\mathrm{i}}\ge 0,\mathrm{ i}=1,\dots ,\mathrm{q},$ and ${\upbeta }_{\mathrm{i}}\ge 0,\mathrm{i}=1,\dots ,\mathrm{p}.$ As stated by Engle [17], the best predictor of the conditional variance in (t + 1) is the weighted average of the long-term variance and the new information proxied by the most recent squared error. In this study, we placed a mean ($\mu$ in Eq. 1) and variance ($\nu$ in Eq. 2) equation to investigate the impact of assigned variables on return and volatilities. Significance of $\upmu$ and $\upnu$ parameters^2^ was used to assess the impact of independent variables. As described in Gabauer [21], following modeling univariate volatilities, for N variables, the time-varying conditional variance–covariance matrix is given by

3 $${\mathrm{H}}_{\mathrm{t}}={\mathrm{K}}_{\mathrm{t}}{\mathrm{R}}_{\mathrm{t}}{\mathrm{K}}_{\mathrm{t}}$$

where ${\mathrm{R}}_{\mathrm{t}}$ and ${\mathrm{K}}_{\mathrm{t}}$ are $\mathrm{N}\times \mathrm{N}$-dimensional matrices of dynamic conditional correlations and conditional variances (univariate GARCH model for each series). By construction, conditional correlations in the model are governed by the information known as a-priori. Engle [18] employs the estimation of dynamic conditional correlations $({\mathrm{R}}_{\mathrm{t}}$) as below

4 $${\mathrm{R}}_{\mathrm{t}}={\left({\mathrm{Q}}_{\mathrm{t}}^{*}\right)}^{-1/2}{\mathrm{Q}}_{\mathrm{t}}{\left({\mathrm{Q}}_{\mathrm{t}}^{*}\right)}^{-1/2}$$

5 $${\mathrm{Q}}_{\mathrm{t}}=\left(1-\mathrm{a}-\mathrm{b}\right)\mathrm{S}+\mathrm{a}{\upepsilon }_{\mathrm{t}-1}\mathrm{ a}{{\upepsilon }^{\mathrm{^{\prime}}}}_{\mathrm{t}-1}+{\mathrm{bQ}}_{\mathrm{t}-1}$$

where ${\mathrm{Q}}_{\mathrm{t}}^{*}$ is a $\mathrm{N }\times \mathrm{ N}$ inverted diagonal matrix with the square root of the diagonal elements of ${\mathrm{Q}}_{\mathrm{t}}$. ${\mathrm{Q}}_{\mathrm{t}}$ and $\mathrm{S}$ are $\mathrm{N}\times \mathrm{N}$-dimensional positive-definite matrices which denote variance–covariance matrices of the conditional and unconditional standardized residuals and $\mathrm{S}\equiv \left[{\mathrm{s}}_{12}\right]$. $\mathrm{a}(\mathrm{\alpha })$ and $\mathrm{b}(\upbeta )$ are nonnegative shock and persistency parameters that hold $\mathrm{a}+\mathrm{b }< 1 \left(\mathrm{\alpha }+\upbeta \le 1\right).$ ${\mathrm{Q}}_{\mathrm{t}}$ and ${\mathrm{R}}_{\mathrm{t}}$ would be time-varying if $\mathrm{a}+\mathrm{b }< 1$ is satisfied.

### Hacker and Hatemi bootstrap causality test

In their seminal study, Toda and Yamamoto [51] account for the establishment of vector autoregression estimation in levels and show the test of general restrictions on the parameter matrices regardless of having an integrated or cointegrated process with arbitrary order. Their model does not necessitate the determination of the order of the integration or the test of cointegration properties of the series. Thus, linear or nonlinear restrictions on the coefficients of a level VAR can be estimated through the Wald criterion and usual chi-square critical values. As the authors stated, for a given *p* order VAR process

6 $${y}_{t}=v+{A}_{1}{y}_{t-1}+\dots +{A}_{p}{y}_{t-p}+\dots +{\varepsilon }_{t}$$

where ${A}_{p}$ is an $n\times n$ matrix of parameters for lag *p* and ${y}_{t}$, $v$ and ${\varepsilon }_{t}$ are n-dimensional vectors; the below augmented VAR ($p+d$) model can be used for testing causality between I(1) series

7 $${y}_{t}=\widehat{v}+{\widehat{A}}_{1}{y}_{t-1}+\dots +{\widehat{A}}_{p}{y}_{t-p}+\dots +{\widehat{A}}_{p+d}{y}_{t-p-d}+{\widehat{\varepsilon }}_{t}$$

Hacker and Hatemi [26] estimate the modified WALD statistic through a bootstrap distribution based on this definition. According to the authors, the original model has drawbacks in the employment of a small sample size; thus, they suggest using a leveraged bootstrap distribution to minimize the size distortions. The MWald tests statistic proposed can be calculated as in Eq. 13 following the definitions below

8 $$Y: = \left( {y_{1} , \ldots ,y_{T} } \right)\, \left( {n \times T} \right)\;\;{\text{matrix}},$$

9 $$\hat{D}: = \left( {\hat{v}, \hat{A}_{1} , \ldots , \hat{A}_{p} , \ldots ,\hat{A}_{p + d} } \right)\, \left( {n \times \left( {1 + n\left( {p + d} \right)} \right)} \right)\;\;{\text{matrix}},$$

10 $$Z_{t} : = \left[ \begin{gathered} 1 \hfill \\ y_{t} \hfill \\ y_{t - 1} \hfill \\ \vdots \hfill \\ y_{t - p - d + 1} \hfill \\ \end{gathered} \right]\;\;\left( {\left( {1 + n\left( {p + d} \right)} \right) \times 1} \right)\;\;{\text{matrix,}}\;{\text{for}}\;\;t = 1, \ldots , T,$$

11 $$Z: = \left( {Z_{0} , \ldots ,Z_{T - 1} } \right) \;\;\;\left( {\left( {1 + n\left( {p + d} \right)} \right) \times T} \right)\;\;{\text{matrix}},$$

12 $$\hat{\delta }: = \left( {\hat{\varepsilon }_{1} , \ldots ,\hat{\varepsilon }_{T} } \right)\;\;\;(n \times T)\;{\text{matrix}},$$

13 $$MWald={\left(C\widehat{\beta }\right)}^{^{\prime}}{\left[C\left({\left({Z}^{^{\prime}}Z\right)}^{-1}\otimes {S}_{U}\right){C}^{^{\prime}}\right]}^{-1}\left(C\widehat{\beta }\right)$$

where $C$ is a $p\times n\left(1+n\left(p+d\right)\right)$ matrix, $\otimes$ is the Kronecker product. The $\beta$ equals $vec(v,{A}_{1}, \dots ,{A}_{p}, {0}_{n\times nd})$ and $\widehat{\beta }=vec\left(\widehat{D}\right)$. Here $vec$ and ${0}_{n\times nd}$ indicate the column-stacking operator and zero matrix possesses $n$ rows ans $n(d)$ columns. ${S}_{U}$ is the variance–covariance matrix of the unrestricted VAR model and equals to ${\widehat{\delta }}_{U}^{^{\prime}}{\times \widehat{\delta }}_{U}$.

### Diebold and Yilmaz volatility spillovers

Diebold and Yilmaz [15] propose a new model to examine economic variables' volatility and return spillovers. Although the model is associated with the vector autoregression analysis, unlike Diebold and Yilmaz [14], Cholesky factor identification is not necessitated to orthogonalize the shocks. To overcome this issue, the authors employ generalized VAR decomposition framework. For a given covariance stationary *p* order VAR process

14 $${y}_{t}=\sum_{i=1}^{p}{\Phi }_{i}{y}_{t-i}+{\varepsilon }_{t}$$

where $\varepsilon \sim (0,\Sigma )$ is a vector of the i.d.d process. When we present this process in moving average form, we end up with the following model

15 $${y}_{t}=\sum_{i=0}^{\infty }{\mathrm{A}}_{i}{\varepsilon }_{t-i}$$

where $N\times N$ coefficient matrices, ${\mathrm{A}}_{i}$ satisfies ${\mathrm{A}}_{i}={\Phi }_{1}{A}_{i-1}+{\Phi }_{2}{x}_{i-2}+\dots + {\Phi }_{p}{A}_{i-p}$. The coefficients in this equation are the key component of the system. Authors utilize the framework of Koop et al. [37] and Pesaran and Shin [42] to enable invariant variance decompositions. The *H*-step-ahead forecast error variance decomposition can be calculated as follows

16 $${\theta }_{ij}^{g}\left(H\right)=\frac{{\sigma }_{jj}^{-1}\sum_{h=0}^{H-1}{({e}_{i}^{^{\prime}}{A}_{h}{\sum e}_{j})}^{2}}{\sum_{h=0}^{H-1}\left({e}_{i}^{^{\prime}}{A}_{h}\sum {A}^{^{\prime}}{e}_{i}\right)} H=\mathrm{1,2},\dots ,$$

where $\Sigma$ is the variance matrix for the residual vector $\varepsilon$ that has a standard deviation of ${\upsigma }_{jj}$ for the *j*th equation. Finally, ${e}_{i}$ is the selection vector that has one in *i*th element and zeroes otherwise. By employing volatility contributions from the procedure above total volatility spillover index is calculated as below

17 $${S}^{g}\left(H\right)=\frac{\sum_{\begin{array}{c}i,j=1\\ i\ne 1\end{array}}^{N}{\widetilde{\theta }}_{ij}^{g}\left(H\right)}{\sum_{i,j=1}^{N}{\widetilde{\theta }}_{ij}^{g}\left(H\right)}\times 100$$

where, $\sum_{j=1}^{N}{\widetilde{\theta }}_{ij}^{g}\left(H\right)=1$ and $\sum_{i,j=1}^{N}{\widetilde{\theta }}_{ij}^{g}\left(H\right)=N$. As for the direction of volatility spillovers among the variables, they can be computed through the normalized elements of the generalized variance decomposition matrix. Thus, the received directional spillovers by variable i from all other variable j.^3^ can be presented as

18 $${S}_{i.}^{g}\left(H\right)=\frac{\sum_{\begin{array}{c}i,j=1\\ i\ne j\end{array}}^{N} {\widetilde{\theta }}_{ji}^{g}\left(H\right)}{\sum_{i,j=1}^{N} {\widetilde{\theta }}_{ji}^{g}\left(H\right)}\times100=\frac{\sum_{\begin{array}{c}i,j=1\\ i\ne j\end{array}}^{N} {\widetilde{\theta }}_{ji}^{g}\left(H\right)}{N}\times100$$

The net pairwise spillovers between variable *i* and *j* are basically the difference between the gross volatility shocks received by *j* from *i* and those received by *i* from *j* can be presented as below

19 $${S}_{ij}^{g}\left(H\right)=\left(\frac{{\widetilde{\theta }}_{ji}^{g}\left(H\right)}{\sum_{i,k=1}^{N}{\widetilde{\theta }}_{ik}^{g}\left(H\right)}-\frac{{\widetilde{\theta }}_{ij}^{g}\left(H\right)}{\sum_{j,k=1}^{N}{\widetilde{\theta }}_{jk}^{g}\left(H\right)}\right)\times100=\left(\frac{{\widetilde{\theta }}_{ji}^{g}\left(H\right){-\widetilde{\theta }}_{ij}^{g}\left(H\right)}{N}\right)\times100$$

## Results and analysis

### Data

In this section, we empirically investigate the relationship between Solactive e-Commerce Index (Solec) and Solactive Electronic Payment Index (Solepmt). Our ultimate objective is to assess the effect of a major health shock on the connectedness between these two markets. We use the COVID19 pandemic as a natural experiment for the purpose. In order to tune fine results, we also consider regional case and death statistics of COVID19. Data for these variables are obtained from WHO database. The regions investigated are America, Europe, and South-East Asia designated hereafter by AMRO, EURO and SEARO. The countries within these regions are grouped under the methodology of the WHO classification (see “Appendix” for the list of countries within each group). For the e-commerce market, recall that Solec index tracks the price development of companies operating in the field. This index contains 40 companies from the e-commerce market. Similarly, the Solepmt index traces price movements of the firms active in the mobile/electronic payment industry and formed by 20 firms. The constituents in both indices generate the majority of their revenue from the core business operations in the corresponding market. Therefore, these companies' stock price (index value) is an appropriate gauge for their market conditions and agents’ expectations. Finance literature shows that equity markets can immediately incorporate and reflect the corresponding information in stock prices. In such cases, the dissemination of information is rapidly impounded in the price of a stock and the subsequent price development is corrected accordingly. Therefore, these price changes can be associated with the expected cash flows and changes in the risk-return preferences of investors. Thus, it is widely accepted that the stock market is a barometer to measure the tension and risk attitude in an economy.

The timeframe of our analysis ranges from November 08, 2018 to February 23, 2021 and consist of 576 daily observations for the Solec and Solepmt variables. We split the analysis period into two equal time intervals: pre-pandemic (November 08, 2018–December 31, 2019) and during pandemic (January 02, 2020–February 23, 2021). In our choice, we follow the declaration of WHO [58] on January 02, 2020 about the cluster of pneumonia cases in the People’s Republic of China. Both time intervals contain an equal length of observations, namely 288 days. We use log price and log return series in respective methods. Econometric tests are executed through GAUSS, E-views and R and Ox-Metrics. The data is obtained from Refinitive Eikon and World Health Organization’s database.

Table 1 presents descriptive statistics of the returns of variables. The mean values for both Solec and Solempt indexes are close to zero. Among the changes in COVID19 cases, the highest average values are observed in Europe and America, and the same observation applies to death statistics. While all averages are around zero, Europe and America exhibit slightly higher mean values than Asia. Standard deviation statistics indicate that both indexes have a similar extent of fluctuations in their return series. The standard deviation statistics of COVID19 cases and deaths align with the findings on mean values. According to both case and death statistics, the highest fluctuations occur in Europe and America. The extent of the volatility in case and death statistics in Asia is considerably lower than that of these two continents. To further examine the mean and variance values of three regions’ COVID19 cases and death statistics, we conducted the test of equality through Anova F and Bartlett tests. Results of COVID19 cases indicated that although mean values are not statistically different, variability of the series possesses a significant difference based on the Anova F and Bartlett tests statistics (0.049 and 375.997, respectively). When it comes to the deaths, we obtained similar results (0.0128 and 636.935, respectively). Results show that although the average case and death statistics do not show a significant difference across the regions, the variability of the values is meaningful from the statistics' standpoint. These statistics show that the pandemic illustrates higher uncertainties in Europe and America than in Asia. This finding can be associated with the policies imposed on these regions. Asia had a previous experience with MERS and SARS with the 2002–2004 pandemic that first broke in China in 2002 and then spread to Hong Kong, Singapore, Taiwan, the Philippines, Vietnam, and at a lower extent to the U.S. and Canada. This may have enabled many countries in Asia to react promptly to the COVID19 pandemic and impose large social distancing measures, including curfews and travel ban restrictions. These faster responses to the new pandemic may have contributed to explaining the stated lower infection and fatality rates compared to western Europe and America. The U.S. and Western Europe tended to be reluctant to react to what later became a major economic and health problem. Looking beyond policies-induced disparities among regions, researchers have also investigated other factors such as social norms, culture and customs, and health pre-dispositions, including genetics and immune systems characteristics of populations. Demographics and weather conditions were also among the potential drivers of the differences across continents examined by researchers. These regional disparities cannot be attributed to a single factor. They are most likely the result of a combination of them. Regarding the shape of the probability distribution of return series, we provide skewness and kurtosis statistics. A normal distribution has a value of zero and three for skewness and kurtosis statistics, respectively. Departures from these values would indicate the presence of nonnormality. Skewness statistics demonstrate that all series are negatively skewed, and each series has a value different than zero. Negative skewness values show that the frequency of higher returns is greater than the lower returns. Similarly, kurtosis values depict departures from normality. Accordingly, it can be stated that each series has a leptokurtic probability distribution. The values of 9.03 and 15.29 are evident that the return distributions of both indexes have fat tails. Jarque–Bera test statistics also confirm the non-normality by rejecting the null hypothesis of Gaussian distribution.

**Table 1:** Descriptive Statistics

	R_SOLEC	R_SOLEPMT	EURO_C	ASIA_C	AMRO_C	EURO_D	ASIA_D	AMRO_D
Mean	0.0014	0.0009	0.0026	0.0009	0.0028	0.0001	0.0000	0.0001
Maximum	0.0868	0.1158	0.2317	0.1211	0.3213	0.0207	0.0158	0.0375
Minimum	− 0.1106	− 0.1434	− 0.3814	− 0.1013	− 0.5195	− 0.0269	− 0.0165	− 0.0429
Std. Dev	0.0174	0.0197	0.0862	0.0296	0.1047	0.0073	0.0020	0.0116
Skewness	− 0.7380	− 1.0360	− 1.1228	− 0.0096	− 0.3435	− 0.3968	− 0.2898	− 0.2299
Kurtosis	9.0316	15.2951	7.0469	4.6970	4.9310	5.9453	41.6374	4.9867
Jarque–Bera	925.40	3731.11	257.04	34.56	50.41	111.65	17,918.19	49.90
Observations	576	576	288	288	288	288	288	288

R_Solec is the log-returns of e-commerce index. Likewise, R_Solepmt is the log-returns of mobile/electronic payment index._C and _D denote the COVID19 cases and death in corresponding regions, respectively

**Fig. 1:**
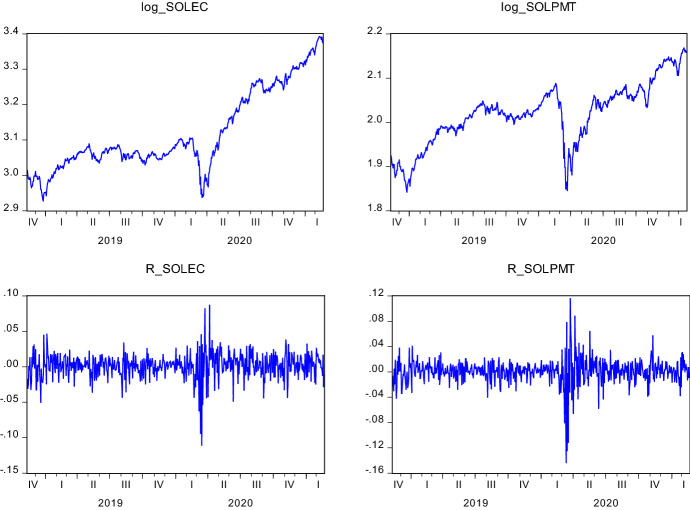
Logarithmic Price and Return Series of SOLEC and SOLEPMT Indices. Solec and Solepmt represents Solactive e-Commerce Index and Solactive Electronic Payment Index, respectively. For log_SOLEC and log_SOLPMT, the y-axis indicates the log prices; for R_SOLEC and R_SOLPMT, the y-axis illustrates the logarithmic price changes of the indices. The x-axis shows the date in each figure in a quarterly scale

**Fig. 2:**
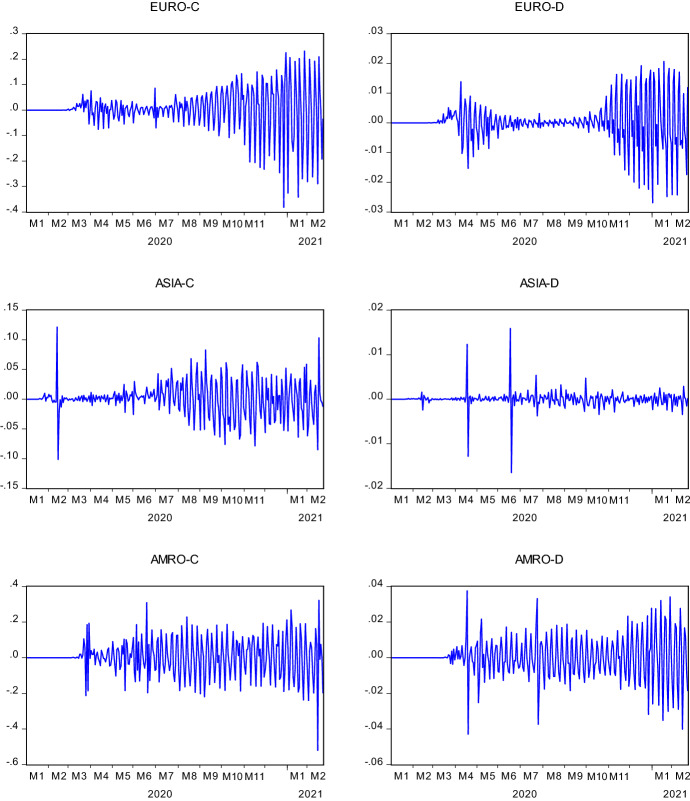
Changes in COVID19 Cases (-C) and Death (-D) in Europe, Asia and America. In the first column, the y-axis indicates the logarithmic changes in regional COVID19 cases; in the second columns, the y-axis indicates the logarithmic changes in regional COVID19 death statistics. The x-axis shows the date in each figure in a monthly scale

Figure 1 illustrates the log price and log return series behavior over the analysis period. Both series display a plunge in February 2020 due to COVID19. The extent of the plummet is seen as more severe in Solepmt variable. Although COVID19 appears to be causing a deterioration in the value of companies that form these two indexes, a swift recovery backed the general upward trend of the log price series. The severe drop is also seen in the fluctuation of the return series. Aligning with the discussion above, the extent of the variability of returns seems wilder in Solepmt index. In Fig. 2, we present the regional case and death statistics of COVID19. According to the changes in cases, both Europe and America exhibit a growing variability over the months. This observation applies to the deaths in America as well. In Asia, however, apart from the marginal changes in the first six months of 2020, death statistics are quite stable. On the other hand, the changes in death statistics of Europe show an entirely different pattern than in other regions and case series. It is evident that there are two waves in this variable. The first wave takes place in April and May 2020. From June 2020 to December 2020, death statistics depict very low variability. However, by starting from November 2020 to February 2021, wild volatility occurs in the behavior of death statistics. This pattern characterizes the severity of the pandemic in this region.

### Empirical investigation

Before proceeding further, we execute a unit root test for the log price and log return series. Employment of nonstationary time series might lead to spurious regression. On the other hand, Hacker–Hatemi bootstrap causality analysis necessitates determining the maximum order of integration of the variables. Therefore, we investigated the stationarity of each series through the ADF and P.P. unit root tests. The results presented in Table 2 suggest that while all return series were stationary, the log price series contained one-unit root, I (1). As the log price data is used in the causality analysis, we will consider this finding when adding the degree of integration to the VAR lag length.

**Table 2:** Unit root test results of the variables

	ADF	PP
L_SOLEC	0.5193	0.3459
R_SOLEC	− 14.7781^***^	− 24.0311^***^
L_SOLEPMT	− 1.4794	− 1.2008
R_SOLEPMT	− 8.8449^***^	− 25.3580^***^
EUROPE_C	− 2.9776^**^	− 18.3893^***^
ASIA_C	− 4.8364^***^	− 17.7172^***^
AMRO_C	− 6.0788^***^	− 23.4704^***^
EUROPE_D	− 4.2115^***^	− 23.3121^***^
ASIA_D	− 15.0146^***^	− 42.7889^***^
AMRO_D	− 6.0788^***^	− 23.4704^***^

The values in the table present the test statistics of both unit root tests. ^**^ and ^***^ denotes statistically significance at the 5% and 1% levels, respectively

As discussed by Engle [18], correlations are one of the essential parameters in financial decisions. From hedge ratio to rainbow options and construction of an optimal portfolio, correlations play a key role in each stage of quantitative modeling. On the other hand, correlations are prone to variations over time and neglecting the dynamic behavior of correlations might bring about less accurate findings in empirical analysis. As stated by Aielli [1], in the dynamic conditional correlation (DCC) model, the conditional variances follow a GARCH process. Thus, time-varying correlations are modeled as peculiar functions of the historical standardized returns in the GARCH model.

**Fig. 3:**
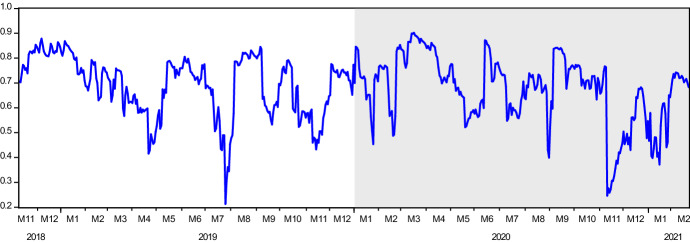
Dynamic Conditional Correlations of SOLEC and SOLEPMT. The y-axis illustrates the time-varying correlation statistics between SOLEC and SOLEPMT indices. The x-axis shows the date in a monthly scale. Shaded area indicates the pandemic period

We present the DCC-GARCH model estimations in Table 3 and dynamic conditional correlations between Solec and Solepmt variables in Fig. 3. The shaded area in the figure displays the period of the pandemic. Results indicate the presence of high historical co-movements between the returns of these two variables. The extent of the correlations does not show a significant difference between pre-pandemic and pandemic days. The dynamic conditional correlations fluctuate between 0.50 and 0.90 most of the time, except for the drops in July 2019 and November 2021. As predicted by the literature, the high volatility in fluctuation is a feature of price and returns evolution during crisis periods [9, 46, 59]. July 2019 marked the culmination of trade-war worries over failed negotiations between the U.S. and China. This led risk-averse investors to look for safer placements found in government securities inducing a sharp drop in stock markets. As for November 2020, financial markets witnessed turbulences over fears of a new COVID19 strain (delta variant) detected in India. The persistence in the high correlation values can be explained by the presence of an initial association between the two markets due to an obvious complementarity in scope and purpose. This association was amplified by the COVID19 outbreak. A 59% percent of the observations depict a higher correlation than the historical average of 0.68. Correlations cannot exceed this value from November 10, 2020, to February 01, 2021.

**Table 3:** DCC-GARCH model for SOLEC and SOLEPMT

	*ρ*	*α*	*β*	df	AIC	SC
Coefficient	0.7057^*^	0.0923^*^	0.8575^*^	6.1425^*^	− 11.85	− 11.77
Std. Error	(0.0478)	(0.0246)	(0.0409)	(0.8057)		

The rho in the table is the coefficient of time varying correlations estimated by DCC-GARCH model

Although correlations are quite useful in determining the direction and strength of the relationship between variables, they are not informative about causality in co-movements. Therefore, we focus on examining the causal link between the variable pairs. We execute the Hacker–Hatemi bootstrap causality analysis. The procedure is based on Toda and Yamamoto [51], who show that an asymptotical distribution accompanied by small samples may cause size distortions, thus, they suggest using a bootstrap distribution. Table 4 contains the results of the Hacker–Hatemi bootstrap causality analysis for the Solec and Solepmt interactions in pre-COVID19 and during COVID19.

**Table 4:** Hacker–Hatemi-J bootstrap causality analysis

	Causality directions			Lag	Selection Criteria	MWALD Test statistic	CV
Pre-COVID19	SOLEPMT → SOLEC			1	[L.R., FPE, AIC, SC, H.Q.]	1.681	6.950 3.853 2.657
	SOLEC → SOLEPMT			1	[L.R., FPE, AIC, SC, H.Q.]	1.986	6.097 3.973 2.866
During COVID19	SOLEPMT → SOLEC			3	[FPE, AIC, H.Q.]	21.33^***^	11.205 8.091 6.330
	SOLEC → SOLEPMT			3	[FPE, AIC, H.Q.]	4.396	12.731 8.015 6.283

^***^denotes significance at the 1% level

In order to calculate the modified Wald test statistics, we follow two steps. First, we form a bivariate VAR model and then, through this system of equations, we determine the optimal lag length that is utilized in the execution of causality analysis. The selection criteria of optimal lag length in each alternative model formation are displayed in the third column of Table 4. Critical values are illustrated in the last column. Results suggest that there is no causal relationship between Solec and Solepmt variables in the pre-COVID19 period. However, a unidirectional causality running from Solepmt to Solec does exist during the COVID19 pandemic at the 1% significance level. This finding postulate that when we were heading for worse times during the pandemic, investors’ positions in these two indexes' constituents also have been corrected. Enthusiasm for online purchasing due to lockdowns and curfews at the onset of the pandemic had not decreased over time. This tendency has been sustained by a high rate of internet penetration along with large efforts by firms to go digital and provide a more user-friendly online purchase experience. The rise in e-commerce's share of global retail translated into higher profitability and higher firm values.

Financial turbulences induced by different factors may generate soaring volatilities. The integration of economies and cross-market linkages expedited the propagation of crisis and transmitted shocks caused severe fluctuations in asset prices. The volatilities and returns can spillover across countries, especially in economic downturn periods, as observed during the COVID19 pandemic and Global Financial Crisis (see [11, 12, 23, 28, 43]). To present evidence regarding this havoc caused by the pandemic in both the e-commerce and mobile/electronic payment market, we test the structural breaks in the volatility of both indices, Solec and Solpmt, through Modified Iterated Cumulative Sums of Squares (M-ICSS) analysis of [44]. Results indicate that both indices contain breaks in the analysis period. The break dates are found for Solec and Solepmt are as follows January 07, 2019, February 21, 2020, and February 25, 2020,and January 09, 2019, February 21, 2020, April 06, 2020, and June 29, 2020, respectively. Our results suggest that both markets display structural breaks that are intensified around the third week of February 2020, which is associated with the plunge in global equity markets due to the pandemic.

In order to detect the direction of causality, we perform the Diebold-Yilmaz volatility spillover analysis to examine the time-varying volatility transmissions. The method is based on the model proposed by Diebold and Yilmaz [14], in which authors utilize forecast error variance decompositions from vector autoregressions (VARs). Unlike the first model, Diebold and Yilmaz's [15] methodology allows variance decompositions not to depend on variable ordering. Additionally, the new approach enables directional spillovers (from/to a particular asset or market) besides the total spillover. Following the authors, we selected the lag length in the VAR model as four days and used a 10-day forecast horizon in the execution of the analysis. The rolling window size is set as 50-day. Directional volatility spillover findings are presented in Table 5.

**Table 5:** Diebold–Yilmaz volatility spillover analysis

	R_SOLEC	R_SOELPMT	From
R_SOLEC	63.89	36.11	36.11
R_SOLEPMT	29.42	70.58	29.42
Directional to others	29.42	36.11	65.53
Net Directional to others	− 6.69	6.69	32.76

**Fig. 4:**
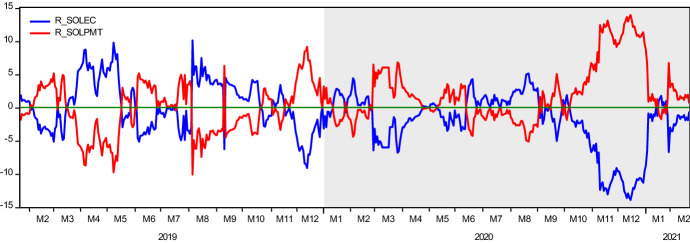
Time-Varying Volatility Spillovers. The y-axis illustrates the time-varying volatility spillovers for both indices, namely, SOLEC and SOLPMT. The values above and below zero show if any of the indices is volatility transmitter to other or receiver from the other, respectively. The x-axis shows the date in a monthly scale

**Fig. 5:**
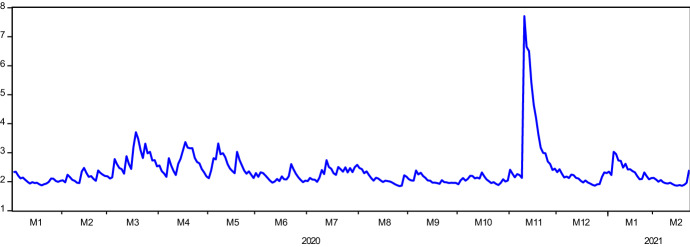
The Change in the Volatility of SOLEC During COVID19. The y-axis illustrates how many times the volatility of Solec index returns during the pandemic is greater than the pre-pandemic period. The x-axis shows the date in a monthly scale

Results in Table 5 show that in terms of the volatility contribution of variables to each other (third row), the Solepmt index appears to be more aggressive than the Solec index. This finding is aligned with the results obtained in the causality analysis. According to the gross directional volatility spillovers received from other variables (last column), as it is expected, Solec is found as a variable receiving higher volatility. Thus, net directional volatility spillovers transmitted to other variables is positive in Solepmt and negative in Solec index. To examine the time-varying behavior of net volatility spillovers transmitted across the years, we provide Fig. 4. In the graph, any value above (below) zero depicts the net volatility transmitter variable (receiver). Accordingly, it is seen that while before the pandemic, the Solec variable was a net volatility transmitter with a ratio of 52%, during the pandemic, along with the varying nature of the interactions as observed in the causality analysis, the Solepmt index turned into net risk transmitter with a radical change in the ratio, from 48 to 61%. Channels of contagion may account for this drastic change in net volatility transmission (Fig. 5). This finding features one of the standard results in the financial literature. The strength of volatility in financial markets rises during turmoil as markets tend to move in tandem during financial crises. During these periods, financial markets' linkages generate higher connectedness which, in turn, feeds stronger volatility.

Diebold-Yilmaz volatility spillover analysis revealed a significant increase in the transmission of uncertainties from the Solepmt index to the Solec index following the pandemic's emergence. The elevated stress in the market is catalyzed by the cases and death statistics from different regions. Although strict measures are imposed to tackle the pandemic, such as travel bans, lockdowns, curfews and social distance practices, the pandemic rapidly spread to every continent by inducing severe and long-lasting damage to economies and social life. However, these harsh restrictions and rigid actions could not prevent the emergence of new waves and variants of the virus. Figure 2 demonstrates this for cases and the death statistics in three regions—Asia, Europe, and America. In this section of the study, by taking into account the previous findings, we model the volatility of Solec index through the GARCH model in pre-COVID19 and during COVID19. Results are presented in Table 6. In both periods, we employ the Solepmt index returns as an independent variable to better account for the volatility or return behavior of the Solec index. Besides, we also use the regional COVID19 cases and death statistics for the same purpose.

**Table 6:** GARCH volatility modeling of SOLEC

	Pre-COVID19		During COVID19		
Cst(M)	0.0011^*^ (0.0007)	− 0.0002 (0.0005)	0.0025^***^ (0.0009)	0.0014^*^ (0.0008)	0.0014^*^ (0.0008)
Cst(V)	0.0630 (0.0436)	0.1857 (0.1897)	0.3244^**^ (0.1430)	0.3061^**^ (0.1355)	0.2795^**^ (0.1312)
ARCH	0.1669^***^ (0.0640)	0.0223 (0.0449)	0.1603^***^ (0.0545)	0.0628 (0.0385)	0.0573 (0.0371)
GARCH	0.8020^***^ (0.0594)	0.7210^***^ (0.2441)	0.7504^***^ (0.0747)	0.7598^***^ (0.0576)	0.7773^***^ (0.0553)
R_SOLEPMT (m)	–	0.8114^***^ (0.0512)	–	0.6362^***^ (0.0599)	0.6408^***^ (0.0583)
EUROPE_D (m)	–	–	–	–	0.2213^**^ (0.1010)
AMERICA_D (m)	–	–	–	–	-0.0486 (0.0562)
ASIA_D (m)	–	–	–	–	-0.4267 (0.3835)
LL	853.859	963.399	753.548	844.402	847.223
AIC	− 5.9018	− 6.6556	− 5.2052	− 5.8292	− 5.8279
SC	− 5.8509	− 6.5920	− 5.1543	− 5.7656	− 5.7262

^*^, ^**^, and ^***^ denote the significance at the 10%, 5% and 1% significance levels, respectively. Cst(M) and Cst(V) indicate constant parameter coefficients for the mean and variance equations. Variables with (m) are taken place in the mean equation. Values inside the parenthesis are the standard errors. _D denotes the death in respective regions. Ln(L) is the log-likelihood value. AIC and SC are the Akaike and Schwarz information criteria, respectively

Results suggest that employment of the Solepmt index increases Akaike and Schwarz information criteria and the coefficient of the variable is found to be statistically significant in mean equations. Accordingly, it can be stated that Solepmt positively impacts the returns of the Solec index. We also utilize the regional COVID19 case and death statistics as explanatory variables in this relationship for the pandemic period. Since COVID19 cases are found insignificant in the volatility and returns of the Solec index, we exclude these findings from the table. When it comes to death statistics, we have seen that only Europe-D (deaths in Europe) is found statistically significant at the 5% level out of three regions. This finding suggests that increasing changes in death statistics in Europe positively affect the returns of the Solec index. This observation has parallel to the result in Becker [6], suggesting that death threats have a critical influence on individuals’ behaviors. Infections' impact, however, can be milder. In particular, if countermeasures such as vaccine campaigns are undertaken in a timely manner and at a large scale. We conjecture that the impact materialized only in Europe may be related to the higher progress of e-commerce in that continent. Recall that the literature found that following the pandemic, e-commerce progressed more in regions where it initially lagged behind. As Europe was only the third-largest continent in terms of e-commerce volumes, after Asia and the U.S., the progress realized may have been stronger. Another important dimension in the explanation may rely on the low informality rate in the European economies. This points to the existence of well-established safety nets, including public health provisions. The existence of such benefits may directly reduce the agent's risk aversion and mitigate the effects of uncertainties in the short run. In turn, this may point to the absence of impact of cases as opposed to deaths.

Finally, we also provide the changes in conditional volatilities of the Solec index. Here the change is calculated as ${\sigma }_{duringCOVID19}^{2}/{\sigma }_{pre-COVID19}^{2}$. Therefore, the figure displays how much volatility has increased during the COVID19 pandemic compared to the pre-pandemic period with equal length. In the figure, November 2020 catches our attention. Although the increase in the volatility is 2.29 × on average by this date, in the second week of November 2020, the average volatility of Solec returns climbed 5.69 × on average. A similar observation is experienced in the dynamic conditional correlations of Solec and Solepmt returns. In the same week, the correlations of these two variables significantly fall. Another interesting observation is the change of net volatility spillover in the Diebold-Yilmaz model in the same period. Unlike these two findings, the net volatility spillover's behavior significantly changed earlier, at the beginning of October 2020, and transmissions of volatility from Solepmt to Solec index considerably increased as of October and November 2020. These market developments experienced can be linked to the turbulences witnessed in financial markets over fears of a new COVID19 strain (Delta variant) first detected in India. Recall that, as argued earlier, standard result in the financial literature stipulates that the strength of volatility in financial markets rises during turmoil periods as markets tend to move in tandem during financial crises. During these periods, financial markets' linkages generate higher connectedness which, in turn, feeds stronger volatility. When we consider these reactions' order, it is clear that the wild increase in the volatility of Solec index and declining correlations occurs after the risk and stress transmission from the electronic payment market to e-commerce market stocks.

## Discussion and implications

### Discussion

This study conducted various statistical analyses for the return and volatility of variables to test the relationship between global mobile/electronic payment and e-commerce markets. The study employed e-commerce (Solec) and mobile/electronic payment (Solepmt) indices from Solactive, a Germany-based index provider. Both indices apply to firms that generate most of their income from the core business operations in the corresponding market segment, namely e-commerce and mobile/electronic payment. To assess the impact of the global pandemic on this relationship, we also employed the regional COVID19 case and deaths for three different regions: America, Europe and South-East Asia. Countries within these regions were selected based on the regional classification of the WHO.

Our analysis starts with determining the time-varying relationship between these two markets. For that purpose, we use the DCC-GARCH methodology. Results show high co-movement between the markets in the analysis period. More than 50% of the observations have a greater correlation value than the historical average of 0.68. This high correlation value may indicate the association of digital operations with global consumption. The relationships between the markets may facilitate the efficiency of tracking systems of the governments in tax collection due to the lessened off-balance sheet records. Although we detected strong co-movements between these two markets based on the DCC-GARCH model, the direction of causality is not yet determined. To validate this result, we used the Hacker–Hatemi-J Bootstrap Causality analysis in two different periods: pre-pandemic and during the pandemic. According to the Hacker–Hatemi-J Bootstrap Causality analysis results, the direction of causality runs from Solepmt to Solec index. Interestingly, this effect seems to be present only during the COVID19 pandemic. This finding aligns with our market observations as well as with studies conducted on consumer behavior for the use of digital technologies (see UNCTAD [52]. Developments that occurred during the pandemic, such as social distancing, lockdowns and increasing risk-aversion of consumers, might significantly impact consumers’ purchase patterns. To confirm this finding, we focus on stress transmissions between e-commerce and mobile/electronic payment markets by using the Diebold-Yilmaz volatility spillover analysis. This method allows us to account for the transmission of shocks between the markets, and similar to the Hacker–Hatemi-J Bootstrap Causality methodology that is based on the vector autoregression analysis. Results obtained from spillovers analysis are aligned with the findings of the causality test. Solepmt has a value of 48% in the transmission of the shocks to the Solec index before the pandemic as the net volatility recipient, which turned into a net volatility transmitter with a ratio of 68%.

The study reveals that the pandemic significantly impacts the relationship between global online/electronic payment and the e-commerce market. However, the tests cannot account for this interaction's characteristics or potential drivers. To assess the potential pandemic impacts, we utilized two variables in this relationship: regional COVID19 cases and death statistics. Considering varying characteristics of consumers based on geographic characteristics and culture, we group the data for three different regions (America, Europe and Asia) and employ it in volatility modeling of Solec index. In this model, first, Solepmt and then regional COVID19 case and death statistics were used as explanatory variables on mean and variance equations. Results show that COVID19 cases do not affect either mean or variance equation. However, besides the Solepmt, out of three regions, only the Europe region displays a statistically significant impact on the mean equation of volatility modeling. We suggest two potential reasons for this finding. On average, the relatively older population of the Europe region may play a significant role in this finding. As stated UNCTAD Report [52], elderly people responded more aggressively in shifting from conventional to online shopping. Likewise, Gunay and Kurtulmuş [24] pointed out that the Europe region became less coordinated in responding to the outbreak and failed in non-discrimination principles and values, especially at the pandemic's beginning. Our output aligns with these findings and reveals that consumer behavior may be linked to its cultural, political, and demographic features during the economically and socially chaotic periods such as a pandemic.

### Practical implications

The panic and turmoil in the market in the recent pandemic accelerated the progress of digital payment systems and operations of e-commerce companies. Although this phenomenon is evident across countries, the direction of the interactions between the two markets, namely, e-commerce and online payments is unclear. This observation prompts the current study to understand the mechanisms driving these interactions for the stakeholders. In this study, we investigate the impact of pandemic and consumer behavior on the relationship between the two markets.

Our empirical investigations show the presence of high co-movements between mobile/electronic payment and e-commerce markets before and during the pandemic. The high correlations of these markets may play a significant role in integrating the systems and providing better solutions for consumers. The rapid technological developments enabled a swift increase in e-commerce and mobile/online payments. The pandemic accelerated this pattern. The high time-varying correlations found in this study suggest that the development of digital systems may increase the efficiency of balance sheet management for financial managers. Reducing the off-balance sheet transactions and keeping better records in operations would increase the working capital management. Additionally, increasing operations in digitalization may offer more traceable accounting practices and lessen the exposure to fraud in the context of auditing. Finally, the participation of more firms in these two markets and the increase in digitization will also heighten the efficiency of governments in collecting taxes to accommodate more traceable accounting activities and fewer operations on the off-balance sheet.

The finding of a causal relationship between the mobile/electronic payment and the e-commerce market can be attributed to changing consumer behavior during the outbreak. Soaring anxiety during the pandemic and measures to prevent contaminations led people to employ alternative payment and purchasing systems. The causality analysis indicates that the shifts in mobile/electronic payment occurred earlier than the developments in the e-commerce market. The rapid change in the payment preferences of consumers might urge e-commerce companies to offer better solutions. The finding implies that changes in consumer behavior and preferences led to growth in the e-commerce market, especially during the pandemic. On the other hand, the pandemic likely induced more consciousness in spending for necessities and then changed the preferences in means of payment, these consumption patterns affected the business to offer suitable services. This observation shows the importance of monitoring the trend shifts in the consumption habits of the consumers for the companies.

Finally, as revealed by our analysis, COVID19-related developments (death statistics) in Europe, rather than in America and Asia, have played a significant role in the relationship between mobile/electronic payment and the e-commerce market. This finding can be attributed to demographic and market structure of Europe. When calculating the median age for each region based on WHO regional classifications, the study shows that among three regions, the Euro region had the highest median age (39.57) compared to America (32.64) and Asia (29.48) as of 2022. This factor may govern consumer behavior in the context of risk-aversion. Additionally, according to the UNCTAD Report [53] Europe was ranked third among three regions in terms of e-commerce market size. A relatively smaller market size may make this region more vulnerable to global market developments. Consequently, the market's reaction may become more significant and severe to the news impact and havoc in the markets, such as the COVID19 pandemic.

## Appendix

**Table:**

AMRO		EURO		SEARO
Anguilla	Jamaica	Albania	Kazakhstan	Bangladesh
Antigua and Barbuda	Martinique	Andorra	Kosovo	Bhutan
Argentina	Mexico	Armenia	Kyrgyzstan	Democratic People's Republic of Korea
Aruba	Montserrat	Austria	Latvia	India
Bahamas	Nicaragua	Azerbaijan	Liechtenstein	Indonesia
Barbados	Panama	Belarus	Lithuania	Maldives
Belize	Paraguay	Belgium	Luxembourg	Myanmar
Bermuda	Peru	Bosnia and Herzegovina	Malta	Nepal
Bolivia	Puerto Rico	Bulgaria	Monaco	Sri Lanka
Bonaire	Saba	Croatia	Montenegro	Thailand
Brazil	Saint Barthélemy	Cyprus	Netherlands	Timor-Leste
British Virgin Islands	Saint Kitts and Nevis	Czechia	North Macedonia	
Canada	Saint Lucia	Denmark	Norway	
Cayman Islands	Saint Martin	Estonia	Poland	
Chile	Saint Pierre and Miquelon	Faroe Islands	Portugal	
Colombia	Saint Vincent and the Grenadines	Finland	Republic of Moldova	
Costa Rica	Sint Eustatius	France	Romania	
Cuba	Sint Maarten	Georgia	Russian Federation	
Curaçao	Suriname	Germany	San Marino	
Dominica	Trinidad and Tobago	Gibraltar	Serbia	
Dominican Republic	Turks and Caicos Islands	Greece	Slovakia	
Ecuador	United States of America	Greenland	Slovenia	
El Salvador	United States Virgin Islands	Guernsey	Spain	
Falkland Islands	Uruguay	Holy See	Sweden	
French Guiana	Venezuela	Hungary	Switzerland	
Grenada		Iceland	Tajikistan	
Guadeloupe		Ireland	The United Kingdom	
Guatemala		Isle of Man	Turkey	
Guyana		Israel	Turkmenistan	
Haiti		Italy	Ukraine	
Honduras		Jersey	Uzbekistan	

## Footnotes

1 Across the methodological presentation, we use this notation to represent both Solec and Solepmt variables in respective sections. The GARCH equation, as a final model of empirical analysis, is populated with explanatory variables that account for both the mean and the variance framework. In this equation, to model the volatility of Solec, we will be using first the Solepmt and later regional COVID19 case and death statistics as independent variables.

2 In empirical analysis these two parameters are demonstrated by Cst(M) and Cst(V) respectively.

3 In this methodology, following the construction of the VAR model, variables *i* and *j* are represented by the Solec and Solepmt indices, respectively.
